# Report on the Effect of Interment of Bodies on the Health of Towns. Ordered by the House of Commons to Be Printed, 14th June, 1842

**Published:** 1843-04

**Authors:** 


### ART. XVIII.

1. *Report on the Effect of Interment of Bodies on the Health of Towns. Ordered by the House of Commons to be printed,* 14*th June*, 1842.

2. *Health of Towns. A Bill for the Improvement of Health in Towns, by removing the Interment of the Dead from their Precincts. Prepared and brought in by* Mr. MACKINNON, Mr. COWPER, *and* Mr. BECKETT, *5th August*, 1842.

3. *Ueber den Einflusz der Verwesungsdünste auf die menschliche Gesundheit, und über die Begrabnissplätze in Medizinisch-politzeilicher Beziehung.* Von Dr. V. A. RIECKE.—*Stuttgart*, 1840. 12mo, pp. 221.

*On the Influence of Putrefactive Emanations on the Health of Man, and on Burial Grounds in their relation to Medical Police.* By Dr. V. A. RIECKE.—*Stuttgart*, 1840.

ON a former occasion we noticed the exposure of the abominations practised in the metropolitan burial-grounds, which Mr. Walker published under the somewhat quaint title of " Gatherings from Grave-yards." Since that time these abominations have been brought more and more into public notice until the last session of parliament, when Mr Mackinnon obtained a parliamentary committee to consider the expediency of framing some legislative enactments, (due respect being paid to the rights of the clergy,) so as to remedy the evils complained of. This committee deemed it expedient to consider the subject under the three following heads: 1. Whether the custom of interments within the precincts of large towns or populous places be injurious to the health of the community? 2. In the event of the injury being proved, what remedies could be suggested? 3. In what manner the remedies ought to be applied, so as not to interfere with vested rights?

The committee report, with regard to the first head of the inquiry, that they cannot arrive at any other conclusion than that the nuisance of interments in large towns, and the injury arising to the health of the community from the practice, are fully proved. The committee, then, recommend, with reference to the second head, that such legislative enactments should be framed as would prevent the interment of the dead in or near the habitations of the living. Under the third head an instructive lesson is taught the medical profession, it being recommended that in framing these enactments, particular attention be paid to the vested interests of the clergy and parish clerks. It appears, however, that there has been a very singular, and we cannot help thinking, an undignified collusion between some of the clergy and the undertakers; the former selling to the undertaker the hatbands and scarves with which they are provided by the friends of the deceased, or, in other words, receiving a sum of money instead of them, termed "a complimentary fee." The evidence of the Bishop of London is as follows:

"3016. Does the fee usually pass as between individual and individual, or does it pass through the undertaker as a part of the funeral charge? I think the practice is not with the middle classes of society to give complimentary fees; they give fittings, but there is a good understanding between the undertaker and the clergymen, generally, as to the fittings. In some instances I have known them amount to pounds; the clergyman's complimentary fee is substituted for the fittings. It depends much upon the class of persons; if a vicar goes, he gets a hatband and scarf, and for that hatband and scarf, ten shillings are placed in the hands of the clergyman."

This is one of the "vested rights;"—to have a hatband and scarf. Some of the statements respecting the condition of our grave-yards are curious. The streets of London, according to Dr. Lynch, stink of the church-yards. That gentleman says:

"2683. We know, (Dr. Lynch, his colleague and assistant,) the symptoms of the dead-damp; it is a peculiar indescribable smell, and any gentleman coming up Parliament street may have remarked it; we had the same smell yesterday, and the same to-day; and when I had got a quarter of a mile from this place, coming along Parliament street, a certain state of the wind will bring it palpable to the nose.

"2684. From whence did the stench come? From the church-yards."

The details elicited by the committee are infinitely disgusting and inexcusably disgraceful. But the evil is working its own cure. People cannot now even bury their dead out of their sight, except by methods the most revolting. A person named Targit, of Southampton, raised a newspaper discussion, because not only had the coffin of his deceased wife been exposed, but it had been broken in, and a portion of the drapery exhibited. Mr. Targit ought to have been thankful that the sexton did not hack his wife and her coffin to pieces, and carry the coffin home in a sack for firewood, as is practised in some of the metropolitan burial-grounds. Mr. Baker, of Leeds, mentions a circumstance of which he was an eye-witness. He was with a sexton while digging a grave. Two coffins were opened, and the fresh bones thrown on the surface, when a by-stander said to Mr. Baker, "Look! these are the skulls of my father and my brother, and the bones of my relations; is not this a bad business?" Mr. Baker adds that the speaker was very much shocked, as well he might. There never could be a reasonable doubt entertained that the mode of interring the dead practised now in England is highly injurious to the public health and offensive to public decency, as stated in the preamble to Mr. Mackinnon's bill. It may be doubted, however, whether that bill grapples fairly with the evil. The main questions are medico-legal, and are comprised in the history of the putrefactive process. For example, the size of a ground, (and of course the expense,) will depend much on the nature of the soil, the depth of the graves, and the period a corpse should remain undisturbed. This period is the time required for complete decomposition, a process occupying from six months to thirty years, according to varying circumstances. These circumstances we shall presently notice, remarking here, that in the report there is much less of that *precise* information respecting them than might have been reasonably expected, and still less reference made to them in the proposed enactment. Clause 2 fixes the distance at which cemeteries shall be permitted, at two miles for the metropolis, and one mile for other cities and towns. Clause 4 forbids the disturbance or excavation of existing burial-grounds for twenty years after the last interment. Clause 5 proposes to fix the number of hours a body shall be permitted to remain unburied. Clause 14 provides that the fences of cemeteries shall be of the height of ten feet; and Clause 31 enacts that no grave shall be opened oftener than twice in four years. These five clauses are all (of thirty-eight in the bill) which refer to the medico-legal portion of the question.

As the work of Dr. Riecke (it is a prize essay) contains a complete review of the whole subject, and as, perchance, the opinion of some of our readers may be requested, we will endeavour to supply them with a short analysis of the practical portion of the essay. The subject is arranged in two divisions, the first being devoted to an examination of the influence of putrefactive emanations on health. Dr. Riecke abundantly proves by numerous positive facts that these emanations are injurious to the health; and, while he acknowledges the correctness of Du Châtelet's observations to the contrary, shows that they are exceptions to the general rule, easily explicable, or that some of the attending and modifying circumstances have been suppressed or not observed. Du Châtelet found that at an establishment at Montfaucon from 10,000 to 12,000 horses, and from 25,000 to 30,000 smaller animals are flayed every year. The stench even to his seasoned nostrils was indescribably horrible, yet the knackers' men and boys were healthy, strong, and long-lived; nor was one of their number attacked by the epidemic cholera when raging around them; as if the abominations in which they lived were rather prophylactic of disease than excitant. Dr. Riecke observes that many of the workmen were born and have lived all their lives on the spot; that in fact they are acclimated; and this explains the whole marvel. Otherwise, we might say with the inhabitants of Walcheren, that malaria is not pernicious, because men and women exist in a malarious atmosphere. The plain truth is that a sufficient habituation of the system to poisons of any description is highly prophylactic. The negro living near marshy swamps is in no more danger from the miasm, which would destroy a European in forty-eight hours, than the vaccinated European is from smallpox. Yet in discussions on contagion and infection, this important fact of habituation is continually overlooked. Dr. Riecke takes other opposing facts brought forward by Orfila, Dunglison, Ollivier, Eisenmann, and others, examines them, and refutes the inferences deduced from them; satisfactorily showing that putrefactive emanations are not salutary nor even capable of curing consumption, as has been gravely asserted.

We cannot follow Dr. Riecke through the numerous observations he has collected from various sources in proof of the proposition that these disgusting emanations are injurious to the health, and often immediately fatal. To our minds the proof is complete; whilst every deduction of the author is confirmed by the facts stated in the report of the parliamentary committee. His observation that animal effluvia give a typhoid type to intermittents is interesting; and also that contagious effluvia may be generated in a body for some time after death. The general conclusions at which our author arrives are principally these: that putrefactive emanations are hurtful in a varying degree as circumstances vary. If highly concentrated they produce apoplexy or immediate death; if less concentrated, headach, debility, loss of appetite, putrid fever, &c.; and that specific poisons are not necessarily destroyed by the putrefactive process.

The second division of the work is devoted to a consideration of burial-grounds. The various modes in which man disposes of his dead are noticed, and the practice of interring in churches and church-yards is traced historically. It appears that so early as the fifteenth century intra-mural sepulture was found to be a nuisance, and was forbidden in Nuremberg, except in churches; and this exception ceased in 1571. In the seventeenth and eighteenth centuries, the evil having increased with the population, practices like those complained of in England were found generally prevalent in France and Germany. The medical profession particularly in France, true to its noble purposes, was the first to war with the prejudices of the times, and to teach the truth not more by precept than example. A Parisian physician had the following epitaph to his memory:

"Simon Pierre, vir pius et probus,
Hic sub dio sepeliri voluit,
Ne mortuus cuiquam noceret,
Qui vivus omnibus profuerat."

At Louvain there is the tomb of a celebrated anatomist, with the following;

"Philippus Verhagen
Med. Dr. et prof.
Partem sui materialem
Hic in cœmeterio condi voluit,
Ne templum dehonestaret
Aut nocivis halitibus inficeret."

The discussions excited by the profession were followed by the prope results. In 1774 the parliament of Toulouse ordered a cemetery to be formed outside the city; Laon, Dôle, Lyons, and other towns imitated the example of Toulouse. A government edict was issued on March 10th, 1776; and in 1780 extra-mural cemeteries were formed at Paris. A Prussian cabinet-order, directing extra-mural sepulture was issued in 1775; the Swedish government in Pomerania took up the matter in 1778; Wirtemburg in 1782. In 1784 the Emperor Joseph carried out the plan begun in 1772 by his predecessor, Maria Theresa, by issuing detailed regulations respecting the mode of interment. In 1786 in Hesse Darmstadt, it was ordered that bodies should be placed in regular rows; the depth of graves was fixed, and also the time when a grave might be reopened. Saxony issued regulations on the subject in 1792; East Friesland in 1818 or 1819.

Specific regulations for the management of cemeteries and the interment of the dead were first issued in France on June 12th, 1804; and afterwards at Aaran (Prussia) in 1808. Although England is forty years behind France in this respect, it is worth knowing that edicts, cabinet-orders, &c. have often been useless. The truth is, there is a vast amount of unmitigated prejudice and superstition in the world respecting the disposal of dead humanity. Priests have always derived their richest harvests from death and the grave, and have invariably been the foremost to denounce all, or, if not all, any effective interference. Dr. Riecke states that in many places in Germany the dead are still interred in church-yards near to dwellings. Napoleon planted the cemetery-police laws of France in the countries he conquered; at Rome, Naples, Madrid; in Portugal, the Rhenish Provinces, and the Netherlands. The Dutch sturdily resisted the innovations; and so their churches, in spite of the Emperor, stunk as abominably as the worst of our own sacred charnel houses. In the province of Tras os Montes (Portugal,) the people pulled down the fences of the closed church-yards that they might get their dead placed within the consecrated inclosure; and in many places in the Peninsula, the corpses already interred in the new cemeteries were disinterred and carried to the churches. In Upper Italy, the Austrian government found great difficulty in compelling obedience to its new rules. At Catania the propriety of extra-mural sepulture was agitated by some enlightened individuals; but they were hotly and successfully opposed by a fraternity of pious Capuchins who had contrived to make the road to heaven through the vaults of their abbey-church; and a most profitable road it was for the proprietors, the toll being heavy. The English clergy generally are too enlightened to be guilty of such selfishness, still there is evidently a tendency to stand upon vested rights and exclusive privileges. Mr. Mackinnon apparently knows this; we meet him, cap in hand, before the clergy, in almost every clause of his bill.

Having gone through his subject historically, Dr. Riecke enters on the practical part. The first question he considers is the distance from the town at which a cemetery should be placed. This has been variously stated, as the following table will show.

French regulations of 1804, at 114 to 130 feet.

Arnsberg in Prussia, not less than 500 paces.
Stralsund in Prussia, ,, 1000 paces.
Sigmaringen, earlier regulations, not less than 500 paces.
,, later regulations, ,, 275 feet
Grand-duchy of Baden ,, 717 feet.
According to the opinion of Gmelin, 1000 to 2000 paces.
,, Mr. Atkinson, half a mile.
,, Dr. Copland and Mr. Walker, 2 miles.

Dr. Riecke observes that emanations are carried farther than people generally suspect. The stench of the carrion-pits at Montfaucon is insupportable at a distance of 6500 feet, and with a favorable wind at double that distance. The stench is even perceptible at four times that distance, or about five miles, under favorable circumstances. There is a church-yard at Stuttgart in which 500 bodies are interred yearly. The graves for adults are six feet and a half (English) deep, and for children from three and two-thirds to five one-third feet. Yet a north-west wind renders the putrefactive emanations arising from the ground perceptible in houses distant from 250 to 300 paces. The distance of a cemetery must vary according to circumstances; as, for example, the cemetery for a small population may be nearer than for a large population. The following are Dr. Riecke's recommendations:

| *No. of Inhabitants.* | *Distance of Cemetery.* |
|---|---|
| 500 to 1000 | a minimum of 150 paces. |
| 1000 to 5000 | 300 paces. |
| above 5000 | 500 paces. (p. 98.) |

Cities increase rapidly; it is therefore right to provide against the approximation of buildings to cemeteries, especially when a city occupies itself in "walking out of town," to use Dr. Copland's bow-bell phrase. The Sigmaringen regulations forbid houses to be built on the ground intervening between the cemetery and the town; those of the Grand-duchy of Baden fix the distance from the cemetery at which houses may be built at 1120 feet. A French imperial decree of March 7th, 1812, orders a minimum distance of 325 feet.

How long ought a grave to remain undisturbed? for the size or cost of a cemetery will be greater or less as this period is shortened or prolonged. It is a common wish of mankind to be at rest after death; to repose, literally, in the grave. A penurious economy or a crowded population is not favorable to the indulgence of such a simple prejudice. In some of the London burial-grounds, bodies are turned out of their last homes before they have even become putrid; and are literally hacked to pieces to make room for the numerous new arrivals. A burial-farce is continually being performed, followed by a one act tragedy. The following is an instance from the Report; Mr. Geo. Whitaker *loquitur:*

"400. I have seen coffins broken in the graves, and shovelled away to make room for fresh comers. And the bodies cut to pieces? Decidedly so. How do you mean? Cut with the spade."

A grave-digger at St. Clement's Lane, does not mince the matter, although he minces his "subjects."

"2360. I have taken children up and moved them within a week after they were buried. Sometimes, you say, you have placed them nowhere? I have done away with them. What do you mean by doing away with them? Breaking the coffins up, and cutting the flesh in bits, and burying it."

That sort of work is disgusting, but it is equally unjust to give the dead the exclusive occupancy of their graves for ever. They must be disturbed at sometime, and, medico-legally, that time is indicated by the termination of the putrefactive process. The feelings of survivors, and, in populous places, the survivors themselves do not continue longer than the time so required. We may observe that decay is complete when the soft parts have entirely mouldered away and disappeared. Bones will remain whole for centuries.* The time occupied by this process and during which the grave ought to remain unopened has been fixed differently by different governments.

| | *Years.* |
|---|---|
| The principality of Lippe | 30 |
| Hesse Darmstadt (1786) | 30 |
| Aaran, Prussia | 25 to 30 |
| Sigmaringen (1834) | 30 |
| do. (1836) | 20 to 25 |
| Frankfort on the Maine | 20 |
| Stralsund, Prussia | 16 |
| Wurtemburg | 18 |
| Milan (1791) | 10 |
| Stuttgart | 10 |
| Munich | 9 |
| France | 5 |

Individuals have also fixed the time differently. Gmelin at from thirty to forty years; Von Wildberg at thirty; Frank, twenty-four to

* It is not long since we witnessed the opening of a stone coffin, whose tenant had during life been an ancient Roman gentleman, and who could not have occupied his narrow residence less than 1400 years. The teeth were all sound, and several of the larger bones scarcely touched by decay. The coffin, it must be observed, was embedded in a clayey soil.

twenty-five; Mr. Walker at seven; Rev. Mr. Tyler at fourteen; and Tagg, a London cemetery proprietor, at twelve years. None makes the time so short as Mr. Mackinnon. The time, however, can be no fixed quantity; for, putrefaction being a chemical process, will, like other chemical processes, be variously influenced as to its duration by varying agencies. Electricity, limited or free access of atmospheric air, temperature, (65° to 100° of Fahr. is most favorable), nature of soil as regards lightness, dryness, chemical composition, &c. all modify the process. A high and dry temperature mummifies bodies, especially those that are lean; water at a low temperature saponifies them, and the more readily if fat; rendering them at the same time very durable. One body putrefies sooner than another under apparently similar circumstances. Corpses of the young decay more quickly than corpses of the old; of women sooner than of men; of fat people sooner than of thin. The disease of which the individual died makes a marked difference; and so also his trade. The sexton at Stuttgart corroborates the opinion of Shakspeare's sexton in Hamlet, that the corpses of tanners decompose the most slowly. The depth of the grave has a considerable influence; the deeper, the colder, and the more wet, and consequently the slower the decay. Besides, insects cannot assist the putrefactive process, if warmth and air cannot penetrate to the corpse. Much clothing and a coffin of hard wood retard the process. It is of the greatest importance, however, to know the septic or antiseptic influence of various soils, and in this part of his subject our author is very minute, quoting a great variety of experiments and observations. Soils differ as well in their chemical composition as in their consistence, capacity to absorb and retain warmth and moisture, to abstract oxygen from the air, &c. Argillaceous, ferruginous, loamy, aluminous soils are unfavorable to putrefaction; so also the soils containing humic acid, as for example, moor-earth and bogs. Ireland furnishes illustrations of this fact from time to time, bodies having been taken out of bogs nearly perfect after many years' immersion. Clayey soils, besides being antiseptic, have the additional disadvantage of becoming deeply fissured in hot dry weather. Sandy, marly, and calcareous soils favour putrefaction. We observe that Colonel Fox took every opportunity of inquiring whether the prejudices of the people would allow of quicklime being put into the coffin, and the witnesses generally answered in the negative. It is curious to find how ancient the practice is, for Frank quotes directions from the Talmud, to the effect that the corpse be covered with quicklime with the object, expressly stated, of accelerating decomposition. The ordinance of the Emperor Joseph directed that the corpse should be inclosed in a sack or shroud only, and the grave filled with quicklime. In Hesse Darmstadt, in 1786, it was ordered that quicklime should be put into the grave, and also at Milan, in 1791.*

This varying influence of soil on the dead has not been overlooked in

* Gypsum has been occasionally used for the preservation of bodies. In the museum of the Yorkshire Philosophical Society, there are two plaster casts of corpses taken out of stone coffins of great antiquity, and which were dug up near York. One had contained the body of a young female. The impressions of the trunk, hips, and thighs, as well as of the sandals in which she had been interred, were very distinct. The teeth were the only remains of the body. Metallic ornaments, as rings and ear-rings, and the iron nails in the soles of the sandals were found in the cast.

*cemeterial* ordinances. According to the Arnsberg regulations, a corpse interred in boggy or clayey soils shall remain undisturbed for from twenty-five to thirty years; in loamy soil twenty years: in calcareous or sandy soils ten years. In the Grand-duchy of Baden, a grave in a clayey soil remains unopened for twenty-five years, in a sandy soil for twenty years. We have seen that in France the prescribed period is five years, and Dr. Riecke very naturally inquires why there is so great a disparity in the regulations of the two countries. He thinks climate may make a difference, and also the custom of stamping the earth down upon the corpse, common in Germany. He comes to the conclusion that, all circumstances considered, the grave of an adult should remain unopened for nine or ten years, and of a child seven or eight years.

Burial-grounds should be well drained, and if with a rocky substratum, the latter should not be too superficial. Waste lands, recommended for funeral purposes by Plato, are often unsuitable and far from being economical; the nature of the soil, extent of inclosure, and distance from the town, &c., may render them very expensive. Elevated cemeteries are good; and the higher the elevation the nearer they may be permitted to the city, as putrefactive gases are lighter than the atmosphere, and ascend. On the contrary, cemeteries below the level of the surrounding houses are very bad. Cemeteries should be so placed as to be easily accessible and not liable to be flooded. The topographical position should be attend to. The Arnsberg regulations recommend that the cemetery be placed to the east or north of the town; the Sigmaringen to the north-east; the Baden to the north or north-east. The reason is obvious. The south and west winds being moister, hold the putrefactive gases in solution more readily than the north or east. If possible a slope to the south should be secured; the cemetery will be drier and warmer, and therefore better; for moderate dryness and warmth promote putrefaction.

The depth of graves has been variously fixed as appears from the following:

| | | | |
|---|---|---|---|
| By France | 4 ft. 10 in. to 6½ ft. | Munich | 6 ft. 7 in. |
| Austria | 6 ft. 2 in., *if lime be used.* | Frankfort on the Maine | 5 ... 7 ... |
| Hesse Darmstadt | 5 ft 7 in. to 6½ ft. | Arnsberg, minimum | 4 ... 7 ... |
| Sigmaringen | 6½ ... | Dr. Copland | 6 to 7 ft. |
| | | Bishop of London | 4 to 5 ... |

The age of the deceased should make a difference in the depth of the grave. The Sigmaringen regulations direct that the coffin of a child shall be four feet below the surface. In the Grand-duchy of Baden, a grave for a person above ten years of age must be five feet seven inches deep; for a child one sixth less. At Aaran six feet nearly for an adult, and five feet for a child. At Stuttgart, and at Glasshütte in the Erzgebirge, the depths are fixed relatively, as follows:

| Glasshütte. | | Stuttgart. | |
|---|---|---|---|
| Individuals under 8 years old | 3 ft. 8 in. | Individuals under 8 years | 3 ft. 9 in. |
| „ from 8 to 14 | 4 ... 7 ... | „ from 8 to 10 | 4 ... 7 ... |
| Adults | 5 ... 6 ... | „ from 10 to 14 | 5 ... 7 ... |
| | | Adults | 6 ... 7 ... |

Dr. Riecke recommends the first three depths of the Stuttgart regulations for children under seven years of age, for youths from seven to fourteen. and for adults, or all above fourteen. [He supports these measurements by satisfactory statements.

To economise the ground, graves should be made in rows according to their depth; children in one row, youth in another, adults in another. Family-graves should be in a distinct part of the cemetery, and arranged according to a fixed plan. These are made a luxury in parts of Germany, and well taxed: or altogether prohibited, as in Sigmaringen, Darmstadt, and Aaran. We agree with Dr. Riecke in thinking that the policy of thus severing family ties and wounding domestic feelings is doubtful, considered morally. And a real loss is experienced by the public in the absence of those elegant funeral monuments which not unusually decorate family tombs.

The size of the grave is fixed by several continental regulations. At Stuttgart the grave of an adult must be six and a half feet by three and a quarter; and an interval of eleven inches nearly between each grave; making a surface for each grave of not quite thirty-one square feet.* At Arnsberg and Munich the measurements are a few inches more or less than the preceding, making altogether a difference of two or three square feet. The French regulations do not give the length; only the breadth, and the thickness of the earthy wall or partition between each grave. The former is fixed at four fifths of a metre, about thirty-one inches; the latter at top and bottom at from eleven and a half inches to nineteen and a half, and at the sides from eleven and a half to fifteen and a half inches. A French grave, according to these measurements occupies a surface of from twenty-four to twenty-seven and a half square feet. The Emperor Joseph's edict fixes the breadth at four feet, and the thickness of the partition at the same, giving a superficies of not less than seventy to eighty square feet. The thickness of the partition wall averages eleven inches in Germany. Dr. Riecke thinks this too little; it should be at least double, or twenty-two inches. In heavy soils the French maximum of nineteen inches might be adopted; but in very light ground the partitions should be from three to four feet thick; and under all circumstances, the deeper the grave the thicker must be the partition. Our author deduces from various data that the length of a grave for an adult, should be eight feet three quarters, nearly; the breadth four feet two thirds; and the whole surface about forty square feet. The grave of an individual aged from seven to fourteen years should occupy a surface of nearly twenty square feet; and if a child, not quite sixteen square feet; the length and breadth for the latter, including the side and end partitions, being two feet nine inches and a half, and five feet seven inches.

We are enabled from the preceding data to calculate the size of a burial-ground for a given population, having a known mortality at various ages. The rule Dr. Riecke proposes is, to multiply the average square superficies of a grave by the average yearly mortality, and again by the number of years a grave should remain unopened, and the product is the measurement required. For example, a cemetery is proposed for a parish having 35,000 inhabitants; the mortality being 1000 per annum, or one in thirty-five. Of the whole number of deaths, one half, or 500, are adults; 450 infants and children under seven years of age; and the remaining 50 ranging from seven to fourteen. Each individual of the

* This is Dr. Riecke's calculation. We do not make it so much.

first class would require forty-eight square (Wurtemberg) feet of surface; each of the second, twenty-four, and each of the third, twenty square feet. Each of the first class would require a decennial grave, of the second an octennial, of the third, a septennial. The square feet required would be

| English feet. | | sq. ft., | | mortality, | | age of gr., | | sq. ft., | | Eng. sq. feet. |
|---|---|---|---|---|---|---|---|---|---|---|
| 54·72 .... | Adults ...... | 48 | × | 500 | × | 10 | = | 240,000 | = | 273,600 |
| 27·36 .... | Youth ...... | 24 | × | 50 | × | 8 | = | 9,600 | = | 10,944 |
| 22·80 .... | Children .... | 20 | × | 450 | × | 7 | = | 63,000 | = | 71,820 |
| | Giving a total superficies of | | | | | | | 312,000 | = | 356,364 sq. feet. |

The advantages of the plan of classifying the interments according to the age of the deceased are here obvious. An average of thirty-five square feet to each corpse, and a decennial grave would raise the required land to a surface of 350,000 square feet; so that the preceding plan economizes 37,400 square feet of ground. Of course, this is only an *average* calculation. Soil and the other circumstances referred to will demand an increase or diminution in the factors. Allowance must also be made for epidemics, and for the increase of population. Walks between each row of graves would be necessary, which, in the instance just mentioned, Dr. Riecke calculates at 58,000 square feet; making a total surface of burial-ground for a fixed population of 35,000, equal to 370,000 square (Wurtemburg) feet, and being about 9·15 square feet (English) to each inhabitant. The Sigmaringen regulations allow thirty square (Wurtemburg) feet, to each inhabitant; in the Grand-duchy of Baden, the allowance is twenty-five square feet; with undisturbed possession for the periods before specified.

The practice of having common graves for the lower classes is of high antiquity. They were termed *pulticuli,* and were used in Rome in the time of Horace.

"*Hoc miseræ plebi stabat commune sepulchrum.*"

Common graves are barbarous things, and cannot be recommended as being either economical or decent. In Paris they became such a horrid nuisance, worse even than they are now in London, that in 1804 they were prohibited altogether. Augustus closed the Roman *pulticuli* for similar reasons. Naples and Trieste have common graves resembling the pulticuli, and a most appalling spectacle do they present.

The free circulation of the air in burial-grounds, should not be checked by too high walls. Ten feet, as Mr. Mackinnon proposes, is we think, an unnecessary height. In France in 1804 it was fixed at six feet and a half; in Sigmaringen very lately, at three feet three quarters. Trees should be planted in groups and not in rows; for in rows they act like high walls. A row, however, would be useful if planted between a burial-ground and closely adjoining houses. Bones should not be collected and placed in catacombs but reinterred. Chinks in clayey soils should be carefully filled with earth. When a cemetery is applied to other purposes, the surface should not be disturbed for at least five years, as directed by the Sigmaringen and French regulations; and if after the lapse of that period it be necessary to dig up the soil, the bones should be collected and reinterred. No wells should be permitted within a certain distance of burial-grounds. The Sigmaringen regulations fix it at 300 feet.

It appears that local inspectors of cemeteries are appointed in Germany, and with great advantage. The inspector should have a plan of his ground, so that he could at any time, accurately identify a corpse. He should also be so acquainted with the duties of his office, that the conduct of details might be safely left to his individual judgment.

The only buildings requisite in a cemetery are, a house for the inspector, a chapel, and a suit of apartments for the reception of the corpses about to be interred.

Dr. Riecke concludes his valuable essay by some judicious remarks on the propriety of so conducting and arranging burial-grounds, that they may be viewed rather as reminiscences of immortality than of death and destruction: and we are happy to say that the new cemeteries in the vicinity of London are all of this description. Well kept, judiciously planted, ornamented with graceful and elegant monuments, and retired from the confusing din of thronged streets, they might be made the best and holiest of temples. In these we hope to have Pére la Chaise improved on. There should be no clusters of nettles; no rotting wreaths of *immortels ;* no neglected graves overgrown with long grass, or "with garden flowers run wild ;" no shattered grave-stones ; no ruined tottering monuments; the hoar of age might be left on them, but nothing more. The feelings touched by Gray's celebrated elegy, and to the touching of these feelings, indeed, its popularity must be partly attributed, are feelings common to all men ; and we believe are capable of such development as to be made no slight auxiliaries to good morals.

With respect to the proposed enactment we would observe, that it is obviously deficient in several essential particulars. This deficiency is perhaps partly attributable to a fear of attempting too much, but mainly, we suspect, to the neglect of Dr. Bowring's judicious recommendation to "gather together the different acts of legislation by which the objects of the bill have been accomplished in other countries, and so ascertain what difficulties have been overcome, and what rights and interests it had been found necessary to recognize." In our review of Dr. Riecke's work we have attempted to supply this want.

We would venture to suggest, as an *economical measure,* the propriety of having inspectors-general appointed who, having the necessary scientific knowledge, might be able to advise and direct the parochial authorities in the choice and size of their ground, and in the formation of their regulations. It would also devolve upon them to guide and control the public taste in the planning and planting of cemeteries, and the construction of funeral monuments. We would also venture to direct attention to the sixth clause of the bill, which constitutes the parson and churchwardens of one parish or more, a "Parochial Committee of Health." As the duties of this committee are simply to provide for the interment of the parochial dead, it is a "Parochial Committee of Burials," and nothing else. Things should be called by their right names. We honour parsons and churchwardens, but we think a committee of health, in the proper sense of the term, would be a laughable affair if they alone formed it.

Mr. Mackinnon may rest assured that he has the good wishes and respect of all the thinking portion (and that is now a large portion) of the medical profession.

Since the above article was printed, we learn that the home-secretary has requested Mr. Chadwick to extend his examination of the evidence on the sanatory condition of the population, to the special evidence on the effects of interment in towns. From the direction of Mr. Chadwick's inquiries it is apparent that he has in view the practicability of the appointment of officers of public health qualified by a professional education; and of charging them with the regulation of the places and modes of interment, and with the various arrangements connected with that practice, necessary for the protection of the public health. We learn that the evils of retaining the dead, often in the single room of the family, for long periods before interment, has been the subject of much inquiry from the medical officers of the unions, and is proved to stand in need of much further examination. The oppressive expense of funerals, and the exorbitant charges of the undertakers, are also subjected to a scrutiny, with the view to amendment. Such of our readers as have seen Mr. Chadwick's published Reports, or who have perused the first article in our present Number, will be prepared for some most important results from his inquiries.

